# Prolonged capillary refill time is associated with intolerance to enteral nutrition in critically ill patients

**DOI:** 10.62675/2965-2774.20260017

**Published:** 2026-09-11

**Authors:** Lucas Vieira de Souza, Magno Tauceda Borges, Gilberto Friedman, Rafael Barberena Moraes

**Affiliations:** 1 Hospital Mãe de Deus Intensive Care Unit Porto Alegre RS Brazil Intensive Care Unit, Hospital Mãe de Deus - Porto Alegre (RS), Brazil.; 2 Universidade Federal do Rio Grande do Sul Porto Alegre RS Brazil Postgraduate Program in Pulmonology Sciences, Universidade Federal do Rio Grande do Sul - Porto Alegre (RS), Brazil.

## INTRODUCTION

Early initiation of enteral nutrition (EN) is associated with better outcomes in critically ill patients.^(1)^ However, in the presence of hemodynamic instability, intolerance to EN is more prevalent and associated with intestinal hypoperfusion.^(2)^ The assessment of peripheral perfusion through capillary refill time (CRT) and its association with visceral hypoperfusion and, therefore, with intolerance to EN, has not been well established.

## METHODS

This is a prospective, observational, single-center cohort study conducted in the intensive care unit (ICU) of *Hospital Mãe de Deus* (Porto Alegre, Brazil), a tertiary hospital with 60 ICU beds (Plataforma Brasil - 75241623.6.0000.5328).

Adult patients who initiated EN during their ICU stay were included. Exclusion criteria comprised imminent death, absolute contraindication to EN (active gastrointestinal bleeding, intestinal obstruction, abdominal compartment syndrome), abdominal surgery within 30 days, acute pancreatitis, and use of parenteral nutrition or oral diet.

Capillary refill time was prolonged when > 3 seconds^(3)^ and was measured at the time of EN initiation (Day 0), as primary exposure, and every 12 hours for 72 hours. Enteral nutrition intolerance was defined by the occurrence of vomiting, diarrhea, abdominal distension, elevated gastric residual volume and/or intestinal ischemia (defined by imaging). The norepinephrine equivalent dose (NED) was also calculated on Day 0.

Based on an expected EN intolerance incidence of 55.7%,^(4)^ an alpha error of 5%, and a power of 80%, the calculated sample size was 104 patients. Student's *t*-test or Mann-Whitney test were used for quantitative variables. Pearson's Chi-squared and/or Fisher's exact test were used to assess associations between qualitative variables and outcomes. A Receiver Operating Characteristic (ROC) curve was generated to assess CRT using intolerance to EN as the gold standard.

## RESULTS

Between January and April 2024, 104 patients were enrolled; 96 (92.3%) tolerated EN, while 8 (7.7%) were intolerant (Table 1S - Supplementary Material). Capillary refill time differed significantly between groups at all evaluated time points. Prior to EN initiation, prolonged CRT was observed in 50% of intolerant patients and 13% of tolerant patients (p = 0.023). When all measurements over 72 hours were considered, prolonged CRT remained more frequent in the intolerant group (75% *versus* 18.8%; p = 0.002) (Figure 1). Analyzing persistently altered CRT, we observed that a cutoff above 40% of altered measurements was the optimal cutoff to identify patients who had intolerance (area under ROC curve = 0.82) (Figure 1S - Supplementary Material).

**Figure 1:**
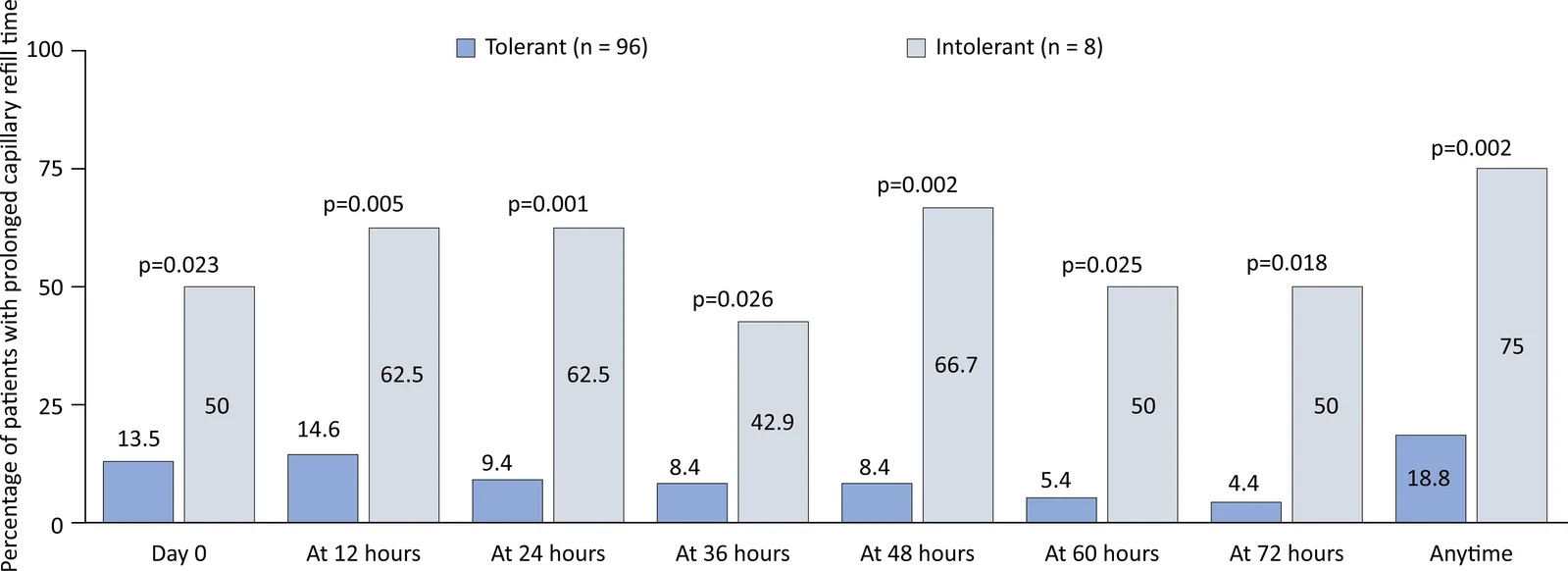
Patients with prolonged capillary refill time according to tolerance at various times of enteral nutrition. Significance level = p < 0.05 by Fisher's test.

Vasopressors were used in 51% of tolerant patients (norepinephrine equivalent dose [NED] 0.08mcg/kg/minute [interquartile range (IQR): 0.04 - 0.13]) and 37% of intolerant patients (NED 0.16mcg/kg/minute [IQR: 0.01 - 0.40]), with no difference (p = 0.454) (Figure 2S - Supplementary Material). There were no differences in ICU or hospital length of stay; however, hospital mortality was higher in the intolerant group (87.5% *versus* 34.4%; p = 0.005) (Table 2S - Supplementary Material).

## DISCUSSION

Assessing tissue perfusion before initiating EN is crucial to ensure tolerance and achieve nutritional goals. Our study demonstrated an association between CRT and digestive complications during EN. Although perfusion-guided EN initiation remains understudied, previous data suggest a close relationship between peripheral and intestinal perfusion. Brunauer et al. showed that visceral Doppler findings correlate with peripheral markers such as CRT, supporting this physiological link.^(5)^ Our study extends these findings to clinical practice, identifying prolonged CRT as a bedside, objective, and cost-free parameter associated with EN intolerance.

While early EN appears safe at lower NED,^(6)^ prior studies did not assess perfusion parameters. The NUTRIREA-2 trial reported more digestive complications among patients receiving EN^(2)^ than among those receiving parenteral nutrition. However, the EN group received a full caloric intake within 24 hours and had a high NED (0.56mcg/kg/minute), without assessment of peripheral perfusion. The NUTRIVAD study found only 0.5% mesenteric ischemia despite a high NED (0.7mcg/kg/minute).^(7)^ The authors emphasized that, while vasopressor dose matters, gradual reduction and improved tissue perfusion are more relevant for safe EN initiation. Consistently, we found no differences in vasopressor use or NED between groups, whereas CRT differed significantly, highlighting perfusion assessment as a key determinant.

Study limitations include a four times lower-than-expected EN intolerance rate. This may be partly explained by the low proportion of patients with shock in our sample, whereas in the study by Wang et al.,^(4)^ which informed our sample size calculation, shock was an inclusion criterion. The low incidence also limited the use of multivariate analysis, compromising the evaluation of confounding factors for both CRT and intolerance to EN. Furthermore, we did not assess factors such as opioid use, chronic gastrointestinal dysfunction, and previous nutritional status. Moreover, this study was conducted at a single center, and the CRT assessors were not blinded to the nutritional tolerance outcomes, limiting our results, which should be interpreted as hypothesis-generating, and effect estimates may lack stability.

## CONCLUSION

In critically ill patients, prolonged capillary refill time is associated with intolerance to EN. It may serve as an additional bedside observational marker, though further adequately powered trials are needed to determine if it should influence clinical decision-making.

## Data Availability

Data is available on demand from referees.
