# Supplementary material for: Prolonged capillary refill time is associated with intolerance to enteral nutrition in critically ill patients
Source: Crit Care Sci. 2026 Sep 11;38:e20260017. doi: 10.62675/2965-2774.20260017 (PMC13690025; doi:10.62675/2965-2774.20260017)
Supplement: Supplementary file 1 [file 2965-2774-ccsci-38-e20260017-Suppl01.pdf]

Critical Care Science
https://doi.org/10.62675/2965-2774.20260017

Prolonged capillary refill time is associated with intolerance to enteral nutrition in critically ill patients

Lucas Vieira de Souza[1,2], Magno Tauceda Borges[1], Gilberto Friedman[2], Rafael Barberena Moraes[2]

METHODS

Definition of enteral nutrition intolerance and pathophysiology

The prevalence of enteral nutrition (EN) intolerance in the intensive care unit (ICU) varies according to the definitions used in the literature. In a recent meta-analysis, Jenkins et al. found a prevalence of 28% of intolerance when considering gastrointestinal symptoms (vomiting, diarrhea, abdominal distension, and gastrointestinal bleeding) and increased gastric residual volume, and 40% when defined by inability to meet the pre-established caloric target.[1] This latter definition may be inadequate due to factors such as fasting for procedures and other institutional issues that lead to cessation of EN instead of intolerance itself, or it may reflect inappropriately high nutritional targets. Our study considered the most accepted concept, since patients with hypoperfusion/shock redistribute blood flow to central organs, diverting splanchnic flow, which can be aggravated by the use of vasopressors and their secondary vasoconstriction. On the other hand, when EN infusion is initiated, there is an increase in intestinal blood flow, increasing mucosal energy expenditure and oxygen requirements. If there is not sufficient increase in output to meet this demand, tissue hypoxia and non-occlusive mesenteric ischemia may occur, which has different presentations, including those mentioned in the definition of intolerance (vomiting, diarrhea, abdominal distension, gastrointestinal bleeding and/or intestinal ischemia) and high mortality.

Capillary refill time assessment methodology

Regarding the assessment of capillary refill time (CRT), it was performed according to a standardized technique from a previous study (ANDROMEDA study). The assessment was measured by applying firm pressure to the ventral surface of the distal phalanx of the right index finger and increasing it until the skin became colorless, then maintaining it for 15 seconds. The time for the skin to return to normal color was recorded with a stopwatch, and a refill time greater than 3 seconds was defined as abnormal. All measurements were performed in a climate-controlled environment (ICU) as soon as the start of EN was indicated, and repeated periodically every 12 hours for the following 72 hours by only two different evaluators, who underwent specific training for this purpose. Any CRT measurement above 3 seconds in the 72 hours evaluated was considered abnormal for the primary outcome.

Circulatory shock definition

Circulatory shock was defined as hypotension requiring fluid resuscitation and/or vasopressor use and lactate > 2mmol.

REFERENCES

1. Jenkins B, Calder PC, Marino LV. A systematic review of the definitions and prevalence of feeding intolerance in critically ill adults. Clin Nutr ESPEN. 2022;49:92-102.

This is an open access article under the CC BY license (https://creativecommons.org/licenses/by/4.0/).

RESULTS

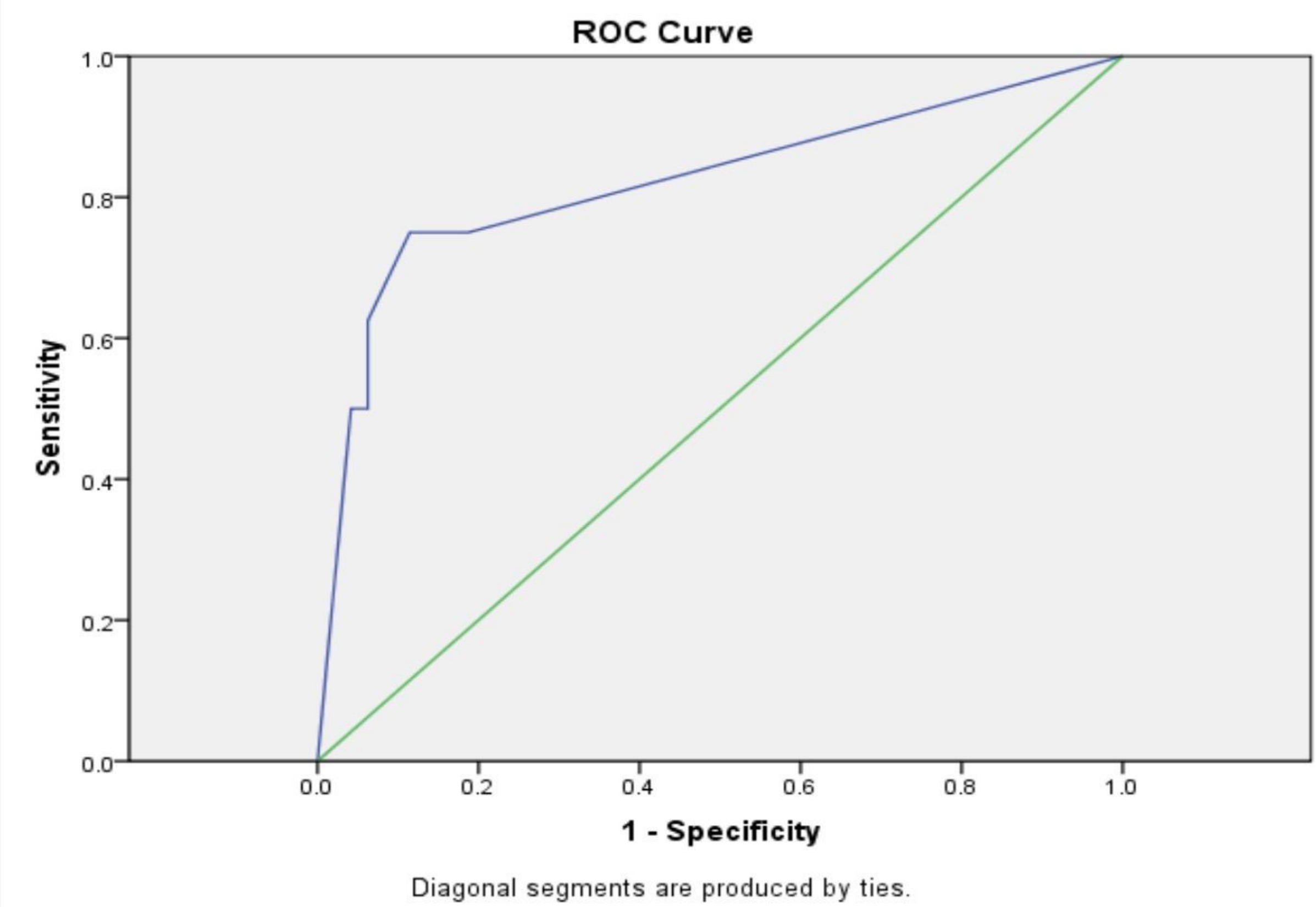

Area Under the Curve = 0.822

Coordinates of the Curve

Test Result Variable(s):TECAL_percentual

Positive if Greater Than or Equal To[a]	Sensitivity	1 - Specificity
-1,0000	1,000	1,000
7,1429	,750	,188
21,4286	,750	,167
35,7143	,750	,115
46,4286	,625	,063
53,5714	,500	,063
68,5714	,500	,052
90,0000	,500	,042
101,0000	,000	,000

The test result variable(s): TECAL_percentual has at least one tie between the positive actual state group and the negative actual state group. a. The smallest cutoff value is the minimum observed test value minus 1, and the largest cutoff value is the maximum observed test value plus 1. All the other cutoff values are the averages of two consecutive ordered observed test values.

ROC - Receiver Operating Characteristic.

Figure 1S - Application of the Receiver Operating Characteristic curve to altered capillary refill time and enteral nutrition intolerance (area under curve = 0.82). Having more than 40% of altered capillary refill time measurements during the assessment period was the optimal cutoff to identify patients who developed enteral nutrition intolerance.

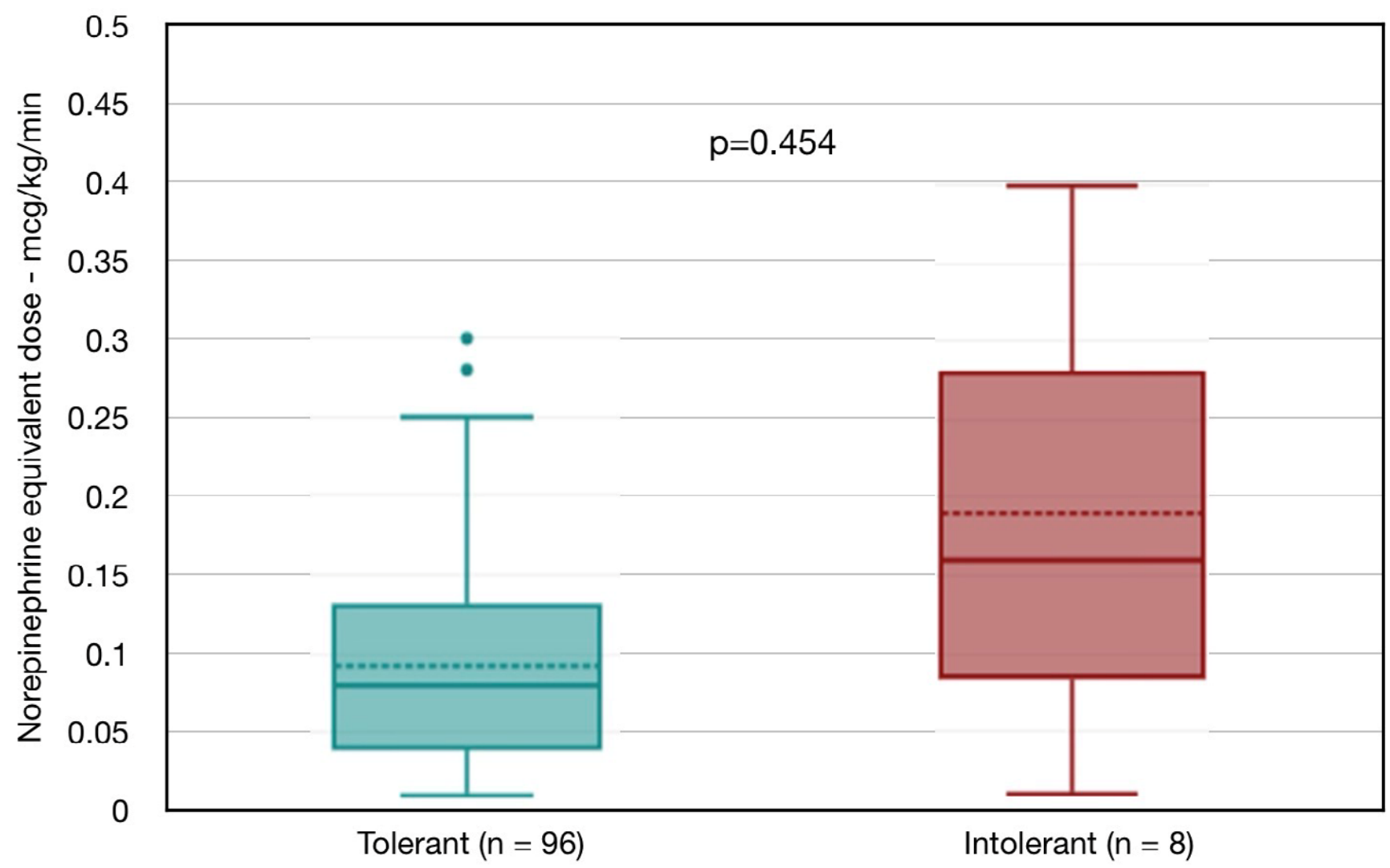

Figure 2S - Comparison of the median epinephrine dose according to tolerance or intolerance to enteral nutrition. Significance level = $p < 0.05$ by Mann-Whitney test.

Table 1S - Characteristics of the patients at baseline

	Total (n = 104)	Tolerant (n = 96)	Intolerant (n = 8)	p value
Age (years)	78 [68 - 86]	78 [68 - 86]	86 [79 - 86]	0.110
Male sex	62 (59.6)	58 (60.4)	4 (50)	0.712
BMI	24.4 [23 - 27]	24.7 [23 - 27]	23.4 [22 - 24]	0.071
SAPS 3	68 [60 - 79]	66 [59 - 79]	70 [68 - 80]	0.155
Comorbidities				
Chronic renal failure	19 (18.3)	16 (16.7)	3 (37.5)	0.159
Liver disease	2 (1.9)	2 (2,1)	0	1
Cardiovascular disease	84 (80)	76 (79.2)	8 (100)	0.348
Chronic pulmonary disease	19 (18.3)	18 (18.8)	1 (12.5)	1
Neurological disease	54 (51.9)	52 (54.2)	2 (25)	0.150
Cancer	24 (23.1)	22 (22.9)	2 (25)	1
Gastrointestinal disease	5 (4.8)	5 (5.2)	0	1
Diabetes	30 (28.8)	29 (30.2)	1 (12.5)	0.433
Acute illness at ICU admission				0.195
Cardiac arrest	3 (2.9)	3 (3.1)	0	
Sepsis	57 (54.8)	52 (54.2)	5 (62.5)	
Acute heart failure or AMI	5 (4.8)	5 (5.2)	0	
Acute CNS failure	22 (21.2)	21 (21.9)	1 (12.5)	
Acute pulmonary failure	2 (1.9)	1 (1)	1 (12.5)	
Trauma	3 (2.9)	2 (2.1)	1 (12.5)	
Acute renal failure	3 (2.9)	3 (3.1)	0	
Postoperative	9 (8.7)	9 (9.4)	0	
Shock	23 (22.1)	20 (20.8)	3 (37.5)	0.371
Sepsis	18 (17.3)	16 (16.7)	2 (25)	
Cardiogenic	4 (3.8)	3 (3.1)	1 (12.5)	
Obstrutive	0	0	0	
Others	1 (1)	1 (1)	0	
Mechanical ventilation	46 (44.2)	41 (42.7)	5 (62.5)	0.461
Mean arterial pressure (mmHg)	82.5 [74 - 93]	82.5 [74 - 93]	82 [72 - 103]	0.971
Heart rate (bpm)	85 [72 - 95]	84 [69 - 95]	87 [78 - 107]	0.329

BMI - body mass index; SAPS - Simplified Acute Physiology Score; ICU - intensive care unit; AMI - acute myocardial infarction; CNS - central nervous system. The Mann-Whitney test was used for the quantitative variables of the groups and Pearson's Chi-squared test and/or Fisher's exact test were used for qualitative variables and the outcome studied. Significance level = $p < 0.05$. Data are expressed as medians [interquartile range] or n (%).

Table 2S - Outcomes

	Tolerant (n = 96)	Intolerant (n = 8)	p value	ARD (95%CI)
Capillary refill time > 3 sec				
Day 0	13 (13.5)	4 (50)	0.023	+36.5 (1.34 - 71.6)
Anytime	18 (18.8)	6 (75)	0.002	+56.2 (22.2 - 90.2)
Vasopressor use	49 (51)	3 (37.5)	0.715	-13.5 (-47.8 - 20.8)
Noradrenaline equivalent dose (mcg/kg/min)	0.08 [0.04 - 0.13]	0.16 [0.01 - 0.4]	0.454	
Lactate (mMol)	1.44 [0.78 - 1.89]	1.67 [1.67 - 1.78]	0.358	
Enteral diet infusion rate (mL/h)				
Day 1	30 [20 - 35]	16 [15 - 20]	0.007	
Day 2	35 [30 - 43]	22 [20 - 24]	0.044	
Day 3	42 [35 - 50]	28 [23 - 33]	0.080	
Caloric density (kcal/mL)				
Day 1	1.5 [1 - 1.5]	1 [1 - 1.5]	0.114	
Day 2	1.5 [1.5 - 1.5]	1.25 [1 - 1.5]	0.184	
Day 3	1.5 [1.5 - 1.5]	1.25 [1 - 1.5]	0.102	
Daily caloric goal				
Day 1	40 [30 - 50]	30 [25 - 38]	0.173	
Day 2	70 [50 - 82]	40 [27 - 57]	0.034	
Day 3	85 [70 - 100]	72 [35 - 88]	0.178	
Use of prokinetics	12 (12.5)	1 (12.5)	1	0 (-18.6 - + 18.6)
Intolerance				
Diarrhea	-	2 (25)		
Vomiting	-	2 (25)		
Abdominal distension	-	2 (25)		
Intestinal ischemia	-	2 (25)		
Length of hospital stay (days)	27 [14 - 40]	18 [11 - 40]	0.526	
Length of ICU stay (days)	8 [5 - 15]	16 [8 - 24]	0.393	
Hospital mortality	33 (34.4)	7 (87.5)	0.005	+53.1 (21 - 85.2)

ARD - absolute risk difference; 95%CI - 95% confidence interval; ICU - intensive care unit. The Mann-Whitney test was used for the quantitative variables of the groups and Pearson's Chi-squared test and/or Fisher's exact test were used for qualitative variables and the outcome studied. Significance level = $p < 0.05$. Data are expressed as medians (interquartile ranges) or n (%).
